# Therapeutic ultrasound and passive mobilisation improve motor function and reduce inflammation in skeletal muscle of immobilised Wistar rats

**DOI:** 10.1002/jeo2.70505

**Published:** 2025-12-28

**Authors:** Fernanda Teixeira Furlan Chico, Ana Caroline Barbosa Retameiro, Aline Reginato, Rafaela Rambo Bremm, Bruna Menegat, Mustafa Munir Mustafa Dahleh, Marina Prigol, Gustavo Petri Guerra, Gladson Ricardo Flor Bertolini, Lucinéia de Fátima Chasko Ribeiro

**Affiliations:** ^1^ Universidade Estadual do Oeste do Paraná Center of Biological and Health Sciences Cascavel Brazil; ^2^ Universidade Federal do Pampa Laboratory for Pharmacological and Toxicological Evaluations Applied to Bioactive Molecules Itaqui Rio Grande do Sul Brazil

**Keywords:** comparative histology, hindlimb immobilisation, morphology, ultrasonic therapy, western immunoblotting

## Abstract

**Purpose:**

Here we analysed the effects of treatment with therapeutic ultrasound, passive mobilisation and their synergically effects on hind paw' functionality, histomorphometric and immunological aspects in the musculoskeletal tissue in an experimental immobilisation model.

**Methods:**

Forty male Wistar rats, aged 10 weeks were divided into (*n* = 8): Immobilised Group, Free remobilisation, Passive Mobilisation, Therapeutic Ultrasound and Passive Mobilisation and Therapeutic Ultrasound. The animals were immobilised, for 21 consecutive days, with a plaster cast orthosis. Therapeutic Ultrasound with frequency of 1.0 MHZ and intensity of 0.5 w/cm^2^ and Passive Mobilisation using degree IV, were performed as treatment. Generalised mixed models and analysis of variance were used for statistical analysis, *p*‐value was set as 0.05. The Immobilised Group had loss in motor function and increase in nociceptive threshold.

**Results:**

Histomorphometry revealed hypotrophy and fibrosis in the muscular tissue, with changes in the muscle fibres, connective tissue and muscle spindles (*p* < 0.0001). Animals that underwent both treatments had the best recovery in those aspects. In the inflammatory tests, the immobilisation caused greater amount of tumour necrosis factor alpha, nuclear factor kappa beta and interleukin‐10 (*p* < 0.05).

**Conclusion:**

Treatments synergistically restored motor function, histomorphometric integrity, and immunological balance in the musculoskeletal tissue of the rats after immobilisation. This may indicate that in humans there should be integration of therapies in post‐immobilisation periods.

**Level of Evidence:**

N/A.

**Abbreviations**
CEUA: Animal's Use Ethics Committee
ERA: effective radiation area
EV: evaluation
FR: free remobilisation
HE: hematoxylin and eosin
IG: Immobilised Group
PM: passive mobilisation
PMTU: passive mobilisation and therapeutic ultrasound
TU: therapeutic ultrasound

## INTRODUCTION

Immobilisation of a limb is commonly employed to reduce pain and prevent further injury by maintaining proper anatomy and supporting structural deformities, usually through casting [26]. Motion at the injury site is limited by this technique, so that healing is promoted and surrounding tissues are protected. However, complications such as pain, swelling, muscle weakness and decreased functionality may be caused by immobilisation, which may result in inactivity. Negative effects, including reduced muscle extensibility and length, as well as increased connective tissue, are produced by muscle inactivity, contributing to muscle hypotrophy [9, 36, 37, 38].

A role in muscle hypotrophy is played by inflammation, with fibroblast differentiation being induced by leucocytes via nuclear factor kappa beta (NF‐Kβ) signalling, leading to muscle fibrosis. Another key cytokine in inflammation is tumour necrosis factor alpha (TNF‐α), by which macrophages, neutrophils and fibrosis are affected [1]. Recovery is complicated by these inflammatory processes, especially after surgery or prolonged bed rest, and mortality can be predicted by muscle weakness [14].

In addition to the inflammatory process caused by immobilisation, it is recognised that several factors can also lead to a similar process of inflammation and hypotrophy. Obesity is associated with increased pro‐inflammatory cytokines and with a contribution to sarcopenia [1]. Aging‐induced sarcopenia itself, as well as chronic diseases such as chronic obstructive pulmonary disease and stroke, are linked to an intensified inflammatory process and to the compromise of muscle tissue integrity, emphasising the need for resources to be employed in these treatments [7], which are often tested in animal models with the aim of reproducing them in humans.

Effective treatment for muscle wasting continues to be considered a challenge due to the limited understanding of its mechanism [1]. Physical rehabilitation, including passive mobilisation (PM) and therapeutic ultrasound (TU), is employed to restore muscle mass and to enhance mobility. Mixed reviews have been reported regarding the effectiveness of PM [14], while tissue repair is facilitated by TU through thermal and mechanical effects, with collagen extensibility and blood circulation being improved [3].

### Aim and hypothesis

In this sense, the functional, histomorphometric, and inflammatory responses of the soleus and plantar muscles in immobilised Wistar rats treated with PM, TU, or both are aimed to be analysed in this study, with the improvement of therapeutic interventions for muscle hypotrophy being sought. The aims of this study were to analyse the functional, histomorphometric, and inflammatory responses of the soleus and plantar muscles in immobilised Wistar rats treated with PM, TU or both. It was hypothesised that synergistic effects on tissue recovery would be produced by the combined therapies.

## METHODS

### Animals and experimental groups

This study is designed as an experimental, randomised, and exploratory research, and has been approved by the Animal Use Ethics Committee of the State University of Western Paraná (Protocol no 09‐21). Ethical guidelines for animal experimentation have been followed in its conduct.

Forty male Wistar rats, 10 weeks old and weighing 278 ± 28 g, were housed at a controlled temperature of 21 ± 1°C with a 12:12 h light‐dark cycle. The rats were assigned to five groups (*n* = 8): Immobilised Group (IG), Free Remobilisation (FR), Passive Mobilisation (PM), Therapeutic Ultrasound (TU) and Passive Mobilisation combined with Therapeutic Ultrasound (PMTU).

A power analysis was conducted based on previous studies in which a moderate to large effect (Cohen's *f* = 0.4–0.6) was expected for histomorphometric and functional outcomes. Using the GPower software, it was estimated that a total sample size of 40 rats (8 animals per group) would be required to detect an effect size of *f* = 0.5 with *α* = 0.05 and power = 0.80.

### Experimental schedule

Initially, a seven‐day adaptation phase was conducted for the FR, TU, PM and PMTU groups prior to the baseline functional evaluation (Evaluation 0, EV0). The baseline assessment was considered essential for outcome comparison after interventions, as it provided a reference point for evaluating changes in muscle function. On the same day as EV0, an immobilisation protocol with plaster casts was applied to all groups for 21 consecutive days [16]. Immediately after cast removal, the IG group was euthanized, and their soleus and plantar muscles were collected to serve as untreated immobilised control samples.

Twenty‐four hours later, the first functional evaluation (EV1) was conducted for the remaining groups, and their respective treatments were initiated: no intervention was applied to the FR group, which remained in free movement; passive mobilisation was administered to the PM group; therapeutic ultrasound was applied to the TU group; and both passive mobilisation and therapeutic ultrasound were administered to the PMTU group.

**Figure 1:**
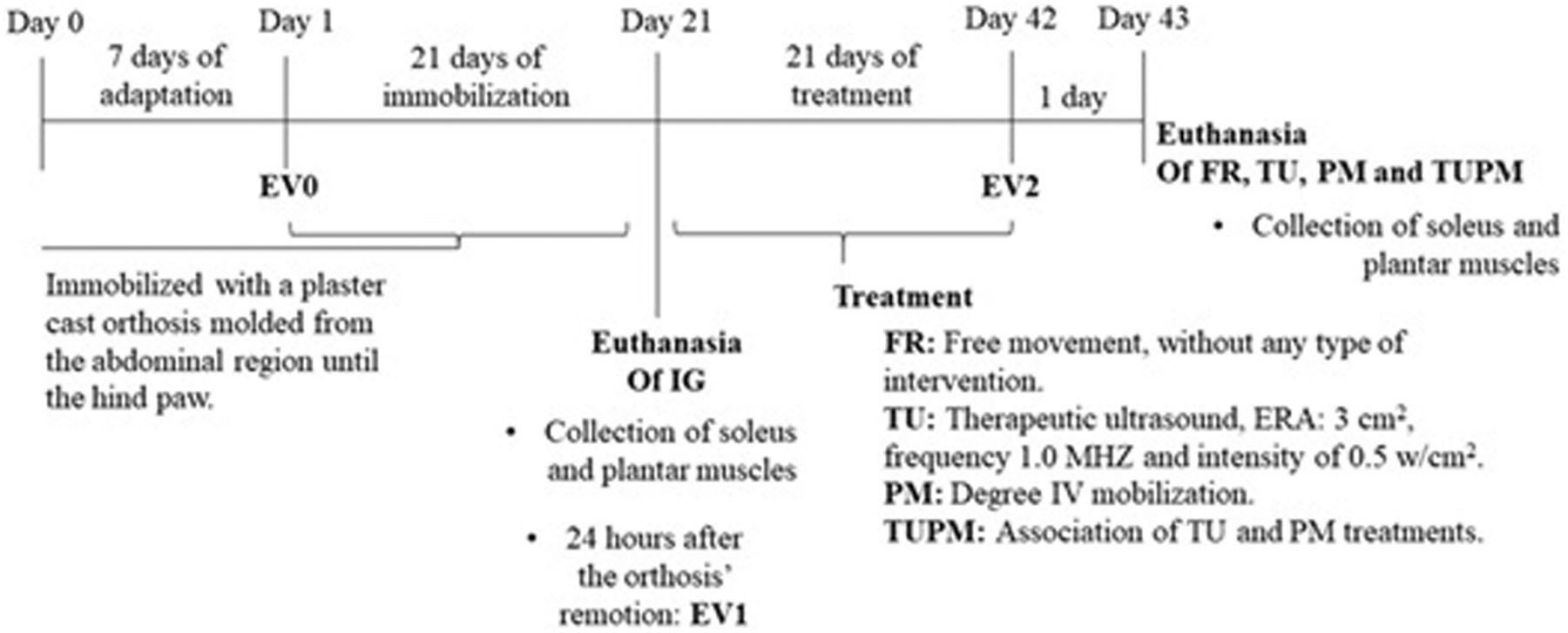
Schematic drawing of the chronological organisation of the experiment (*n* = 8). Are indicated: Immobilised Group (IG); Free Remobilisation (FR); Passive Mobilisation (PM); Therapeutic Ultrasound (TU); Therapeutic Ultrasound and Passive Mobilisation (TUPM); Evaluation 0 (EV0); Evaluation 1 (EV1) and Evaluation 2 (EV2).

In the final week of treatment, a second functional evaluation (EV2) was conducted. Multiple functional assessments (EV1 and EV2) were performed. After the 21‐day intervention was completed, the animals were euthanized 24 h post‐EV2, and soleus and plantar muscle samples were collected for histomorphometric and molecular analyses (Figure 1).

Euthanasia of the IG group immediately after cast removal was performed to provide a control for immobilisation‐induced damage, while the collection of samples from the treated groups after 21 days of intervention allowed recovery to be assessed. Soleus and plantar muscles were collected for histomorphometric and molecular analyses, enabling a comprehensive evaluation of structural and inflammatory changes.

### Immobilisation protocol

The experimental immobilisation model was performed as described by Zazula et al. [40]. After anaesthesia was induced (Ketamine: 95 mg/kg; Xylazine: 12 mg/kg, intraperitoneal), the animals were immobilised for 21 consecutive days (Figure 1) using a plaster cast orthosis molded from the abdominal region, just below the rib cage and extending to the right hind paw. The hip joint was maintained in a neutral position, the knee joint was kept in full extension, and the tibio‐tarsal joint was held in maximum plantar flexion.

### Treatment protocols

#### Passive mobilisation

In the PM and PMTU groups, the passive mobilisation protocol was applied to the right hind paw using a grade IV mobilisation, a small‐amplitude movement reaching the restrictive barrier. In the knee joint, the movement was performed posteroanteriorly, and in the ankle joint, anteroposteriorly, always by the same researcher, in order to avoid bias about the direction and force applied. Approximately 50 movements were performed within 1 min, three times per week, for 3 weeks, totalling nine sessions for both joints (Figure 1).

#### TU protocol

For the TU and PMTU groups, treatment with therapeutic ultrasound was administered using a Sonoplus Ibramed device, with an effective radiation area (ERA) of 3 cm², a frequency of 1.0 MHz, and an intensity of 0.5 W/cm² in continuous emission. Ultrasound was applied for 2 min on the knee (1 min on the lateral face and 1 min on the medial side) and for 2 min on the ankle (1 min on the lateral side and 1 min on the medial side) of the right hind paw. Treatments were conducted three times per week for 3 weeks, totalling nine sessions (Figure 1).

Therapeutic ultrasound parameters were selected based on their suitability for superficial muscle tissues in rats and on alignment with established protocols. Tissues 1–2 cm deep, including the soleus and plantar muscles, are reached by the 1.0 MHz frequency, promoting tissue repair. Safe non‐thermal effects, such as increased blood flow and collagen extensibility, are ensured by the 0.5 W/cm² intensity, while recovery is assessed through the schedule of nine sessions over 3 weeks, consistent with previous studies [16].

### Functional evaluations of the treated groups

#### Animal training and evaluation

Animals in the FR, PM, TU and PMTU groups underwent a seven‐day training period for the functional equipment. Evaluations were conducted on the first day before immobilisation (EV0), after orthosis removal (EV1), and following the third week of treatment (EV2). Results were recorded as the averages of two consecutive trials.

#### Motor function evaluation

Inclined plane test: The animal's ability to remain on a ramp, which was inclined by 5° increments every 5 s, was measured. The test score was determined as the maximum angle at which the animal could remain on the ramp for at least 5 s. Animals were positioned in the transverse plane (vertical position).

#### Nociceptive threshold

*Von Frey* filament test: Mechanical allodynia was assessed using a digital analgesimeter, with evaluation transducer capacity between 0.1 and 1000 g. The animal was placed in a mesh‐floored box, and a filament was applied perpendicularly to the plantar region of the hind paw. Pressure was gradually increased until limb withdrawal occurred, indicating the mechanical allodynia threshold (g).

### Collection and euthanasia

The IG animals were euthanized immediately after orthosis removal, while the FR, PM, TU and PMTU groups were euthanized after 21 days of treatment. Euthanasia was performed via an overdose of ketamine and xylazine. Soleus muscles were collected and fixed in methacarn for histomorphometric analysis. Plantar muscles were also collected, cryopreserved, and stored in an ultrafreezer at −80°C for molecular analysis (Figure 1).

### Light microscopy

#### Sample preparation

Soleus muscles from the right paw were collected and fixed in methacarn solution (70% methanol, 20% chloroform and 10% glacial acetic acid). The samples were dehydrated through an ascending alcohol series, diaphanized with *n*‐butyl alcohol, and embedded in paraffin.

#### Sectioning and staining

Transversal sections (7 μm) were cut using an Olympus microtome (R CUT 4055, Tokyo, Japan). Sections were stained with the following: Hematoxylin and eosin (HE) for evaluation of general histomorphometric aspects; Masson's Trichrome for quantification of general connective tissue; and Picrosirius Red for assessment of collagen types.

#### Microscopy

Histological slides were examined and photographed using a light microscope (Olympus DP71, Tokyo, Japan) and a polarised light microscope (Zeiss Axio Scope A1, Zeiss Industrial Metrology, Germany).

### Morphological and histomorphometrical evaluation of the soleus muscles

Morphological analysis was conducted to assess the effects of inflammation on muscle tissue, using patterns based on pathological changes adapted from by Zazula et al. [40]. Two values were used for histopathological assessment: Importance Factor (*w*), which measures the pathological significance of the change (1 ‐ Minimal, 2 ‐ Moderate, 3 – Severe). Score value (*a*): Reflects the extent of the change (0 ‐ None, 2 ‐ Minimal, 4 ‐ Moderate, 6 ‐ Wide). The change index (*x* = *a* × *w*) was calculated for each change. The total damage index was determined by summing all change indices.

For histomorphometric analysis, the soleus muscle was fixed in methacarn and stained with H/E, Masson's Trichrome, and Picrosirius Red.

H/E staining: Quantified nuclei, capillaries, nuclei‐fibre ratio and capillary‐fibre ratio in 10 fields per animal. Ten muscle fibres per field were measured for area, primary diameter and minor diameter.

Masson's trichrome staining: Analysed connective tissue density in 10 photomicrographs by calculating the percentage of connective tissue pixels relative to total pixels. Muscle spindles were assessed for capsule thickness, diameter, area and number of intrafusal fibers.

Picrosirius red staining: Quantified collagen types by colour (type I in red, type III in green) in 10 fields per animal, expressed as a percentage of total collagen in each field.

All measurements were performed using the Image‐Pro Plus 6.0 programme and GNU Image Manipulation Programme (GIMP 2.10.34).

### Western blotting

Western blot analysis was conducted. For sample preparation, plantar muscles from the rats were dissected and homogenised in 300 μL of ice‐cold buffer A containing various protease inhibitors and salts. The homogenised samples were incubated for 15 minutes at 0°C and then centrifuged at 16,000*g* for 45 min at 4°C. The supernatant, containing cytosolic proteins, was collected, and protein concentration was determined using the Bradford method. Protein extracts were diluted to 80 µg and separated by 12% SDS‐PAGE for approximately 2 h.

The gel was transferred to Amersham™ Protran® Premium Western blotting nitrocellulose membranes using the Thermo‐Fischer® HEP‐1 Transfer System (400 mA for 2 hours). After blocking with 1% BSA in TBS‐T for 2 h, the blots were incubated overnight with primary antibodies: anti‐mouse β‐actin (1:1000), anti‐rabbit NFκB (1:1000), anti‐mouse TNFα (1:500), anti‐mouse IL10 (1:500) and anti‐mouse IL1β (1:500).

The blots were washed with TBS‐T and incubated with secondary antibodies conjugated with horseradish peroxidase (1:5000) for 2 h at room temperature. Development of the blots was performed using TMB, followed by scanning and quantification with ImageJ software. Results were normalised by comparison of the protein bands to the β‐actin loading control.

### Statistical analysis

Results were expressed as mean ± SD for all parameters. For functional evaluations (inclined plane and Von Frey), a mixed generalised model test with Fisher's least significant difference (LSD) post hoc test was used. For morphological and histomorphometric variables, a mixed generalised model test with Sidak post hoc test was employed. These analyses were performed using the Statistical Package for the Social Sciences (SPSS). For molecular analyses, data were assessed using analysis of variance (ANOVA) with Tukey's post hoc test, conducted with GraphPad Prism™ 8.4.3. Statistical significance was set at *p* = 0.05.

## RESULTS

### Functional tests of the treated groups

#### Motor function evaluation

**Figure 2:**
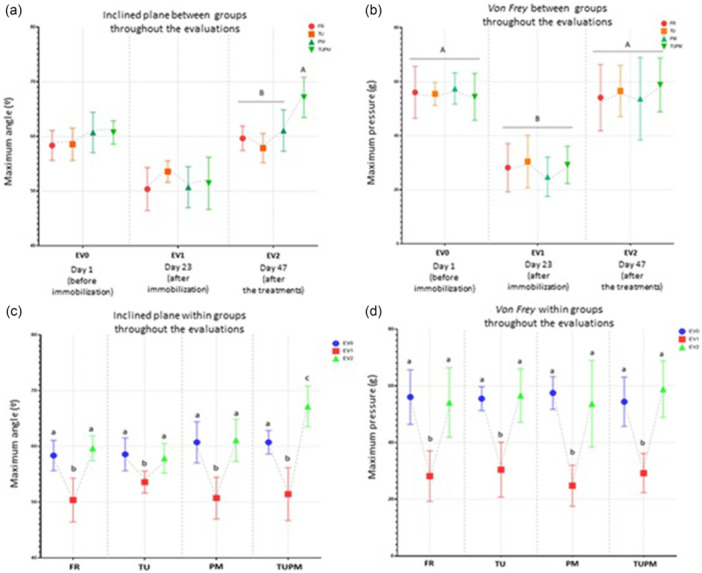
Functional tests results. Free remobilisation (FR), Therapeutic Ultrasound (TU), Passive Mobilisation (PM), Therapeutic Ultrasound and Passive Mobilisation (TUPM), Evaluation 0 (EV0), Evaluation (EV1) and Evaluation 2 (EV2). Different letters represent different values. Values are expressed as mean ± SD. A mixed generalised model test with Fisher (LSD) was used (*p* = 0.05) (*n* = 8). (a, b) Functional tests between groups throughout the evaluations. (c, d) Functional tests results within groups throughout evaluations. (a) and (c) Maximum angle reached by animals during the evaluations in the inclined plane (°). (b) and (d) Maximum pressure supported by the animals during the Von Frey test (g).

In the motor function evaluation, which was performed using the inclined plane test, significant differences were observed with interaction between these factors was detected (*p* = 0.002) (Figure 2a–c). At EV2, TUPM differed from FR (*p* = 0.00006), PM (*p* = 0.001), and TU (*p* < 0.0001), presenting higher values (Figure 2a). Within groups across the evaluations, a decrease in the maximum angle reached at EV1 was observed compared to EV0 (FR: *p* = 0.00002; PM and TUPM: *p* < 0.00001; TU: *p* = 0.006) and to EV2 (FR, PM, and TUPM: *p* < 0.0001; TU: *p* = 0.017) (Figure 2c).

#### Nociceptive threshold

In the nociceptive threshold assessment, significant differences were observed between evaluations (*p* < 0.0001) (Figure 2b,d), while no differences between groups (*p* = 0.846) (Figure 2b,d), and no interaction (*p* = 0.887).

### Analyses performed on cross sections of the soleus muscle stained with H&E

#### Morphological analysis

**Figure 3:**
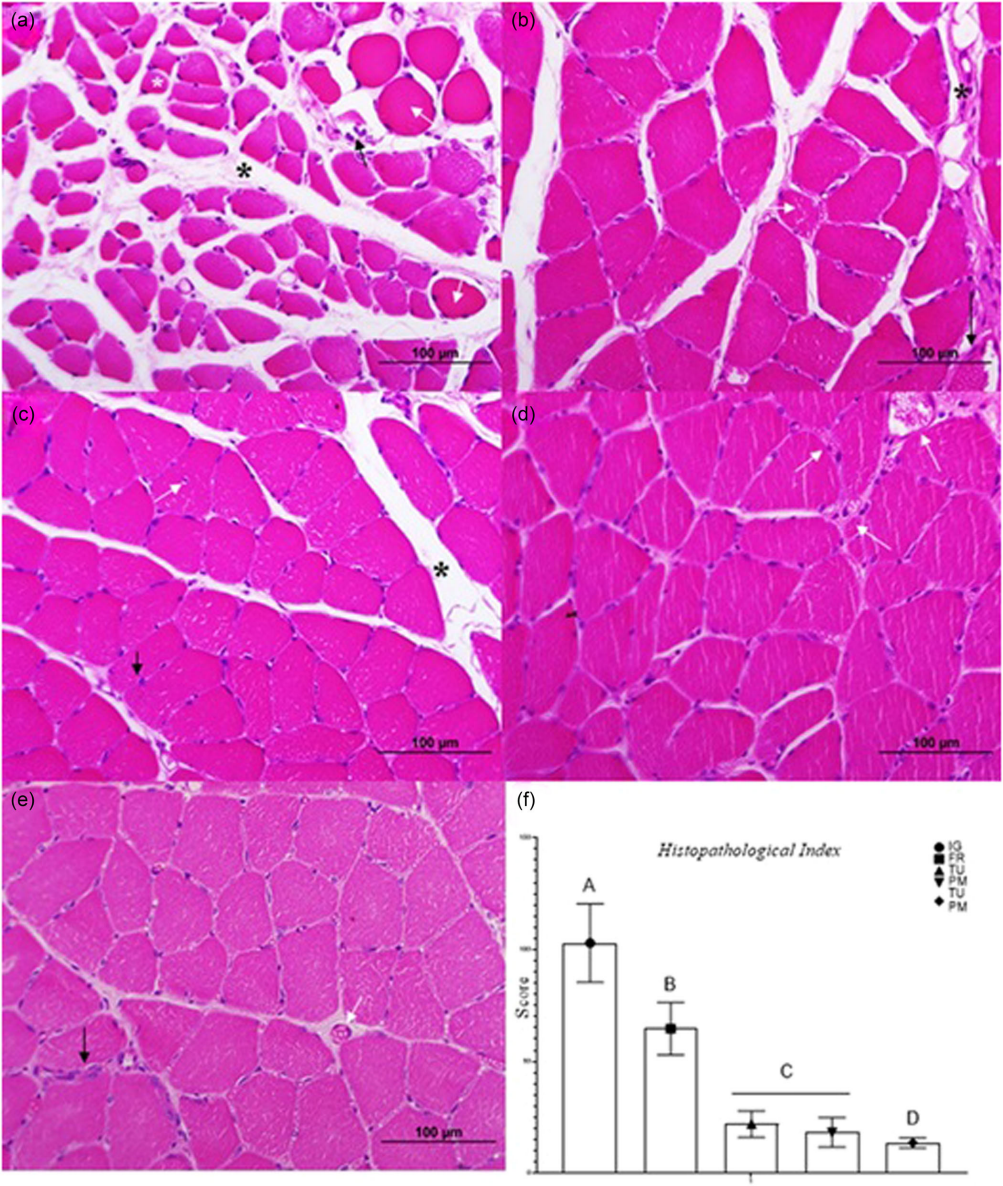
Photomicrographs of the soleus muscle of Wistar rats, with experimental immobilisation, in cross section stained in haematoxylin and eosin (HE). (a) Immobilised Group (IG) with hypertrophy and disorganisation of the connective tissue (black asterisk), fibre hypotrophy (white asterisk), amorphous and hypereosinophilic fibers (white arrow) and tissue necrosis (black arrow). (b) Free Remobilisation (FR) shows congested vessels (white arrow), muscular hypotrophy (black arrow), connective tissue disorganisation and hypertrophy (black asterisk). (c) Passive Mobilisation (PM) presenting centralised nuclei (white arrow), rounded fibers (black arrow) and hypertrophy in the connective tissue (black asterisk). (d) Therapeutic Ultrasound (TU) stood out in the greater amount of blood vessels (white arrow). (e) Therapeutic Ultrasound and Passive Mobilisation (TUPM), in which was possible to notice nuclei centralisation in the fibre (black arrow) and presence of blood vessels (white arrow). (f) Averages of damage in the soleus tissue. Different letters represent different values. Values are expressed as mean ± SD. A mixed generalised model with Fisher (LSD) post‐test was used (*p* = 0.05) (*n* = 8).

In the qualitative morphological analysis, amorphous, atrophic, vacuolated, and hypereosinophilic fibers, as well as some fibers undergoing necrosis, were observed in the IG group. In addition, inflammatory infiltrate was detected in the tissue, and hypertrophy and disorganisation of the connective tissue were noted (Figure 3a,f).

Regarding the treated groups, congested blood vessels were observed in the FR group, in addition to tissue disorganisation, connective tissue hypertrophy, and atrophied fibers, although at a lower density than in the IG group (Figure 3b,f). In the PM group, a small amount of disorganised connective tissue, some rounded and amorphous fibers, and centralised nuclei were observed (Figure 3c,f). In the TU group, a large number of blood vessels were detected, indicating increased vascularisation and blood congestion (Figure 3d,f). In contrast, the TUPM group exhibited the best tissue organisation, although some nuclear agglomerations and congested vessels were still present (Figure 3e,f).

In the quantitative assessment of histopathology, the characteristics observed in the qualitative morphological analysis were confirmed (*p* < 0.0001). The highest score was obtained by the IG group when compared to all treated groups (PM, TU and PMTU: *p* < 0.0001; FR: *p* = 0.001), followed by the FR group. PM and TU were not significantly different from each other (*p* = 0.171) and presented lower values compared to IG and FR (*p* < 0.0001). The lowest histopathological value was observed in the TUPM group (Figure 3f).

#### General morphometrical analysis

**Figure 4:**
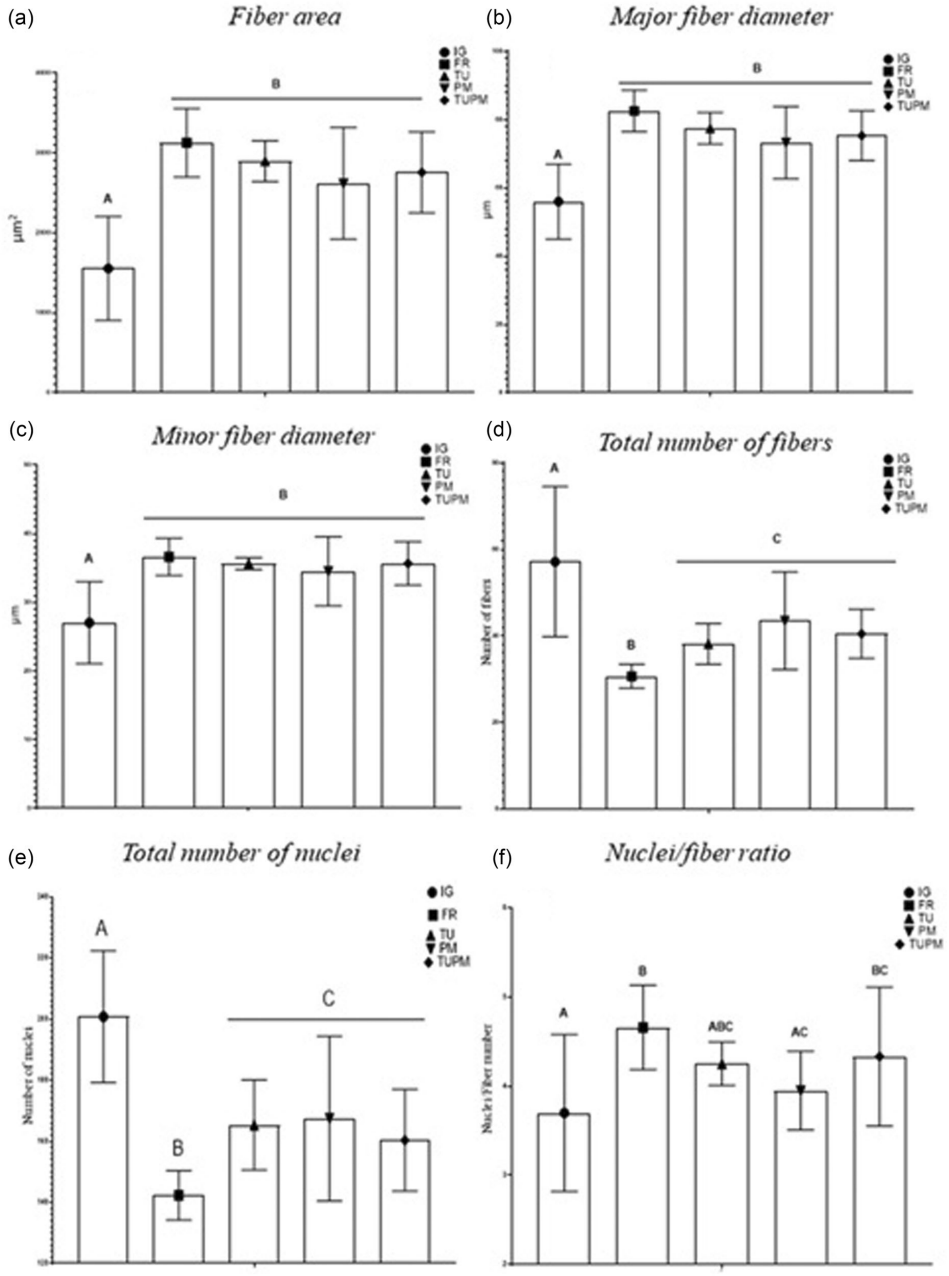
Morphometrical analysis of the soleus muscle results. Immobilised Group (IG), Free Remobilisation (FR), Therapeutic Ultrasound (TU), Passive Mobilisation (PM) and Therapeutic Ultrasound and Passive Mobilisation (TUPM). Different letters represent different values. Values are expressed as mean ± SD. A mixed generalised model test with Fisher (LSD) post‐test was used (*p* = 0.0.05) (*n* = 8). (a) Total fibre area. (b) Major fibre diameter. (c) Minor fibre diameter. (d) Total number of fibers. (e) Total number of nuclei. (f) Nuclei/fibre ratio.

In the morphometric analysis of fibre area (Figure 4a), major diameter (*p* < 0.0001) (Figure 4b), and minor diameter (*p* = 0.00001) (Figure 4c), lower values were observed in the IG group compared to all treated groups. Regarding the number of fibers (Figure 4d), the highest count was observed in the IG group. In contrast, the lowest value was observed in the FR group.

In the nuclei count (*p* < 0.0001), the largest number was observed in the IG group. Conversely, the lowest values were observed in the FR group (Figure 4e). Analysis of the nuclei/fibre ratio (Figure 4f) revealed the highest values in the FR group, while the lowest values were observed in the IG group (*p* = 0.049).

### Analyses performed on cross sections of the soleus muscle stained with Masson's trichrome

#### Muscle spindle morphology and morphometry

**Figure 5:**
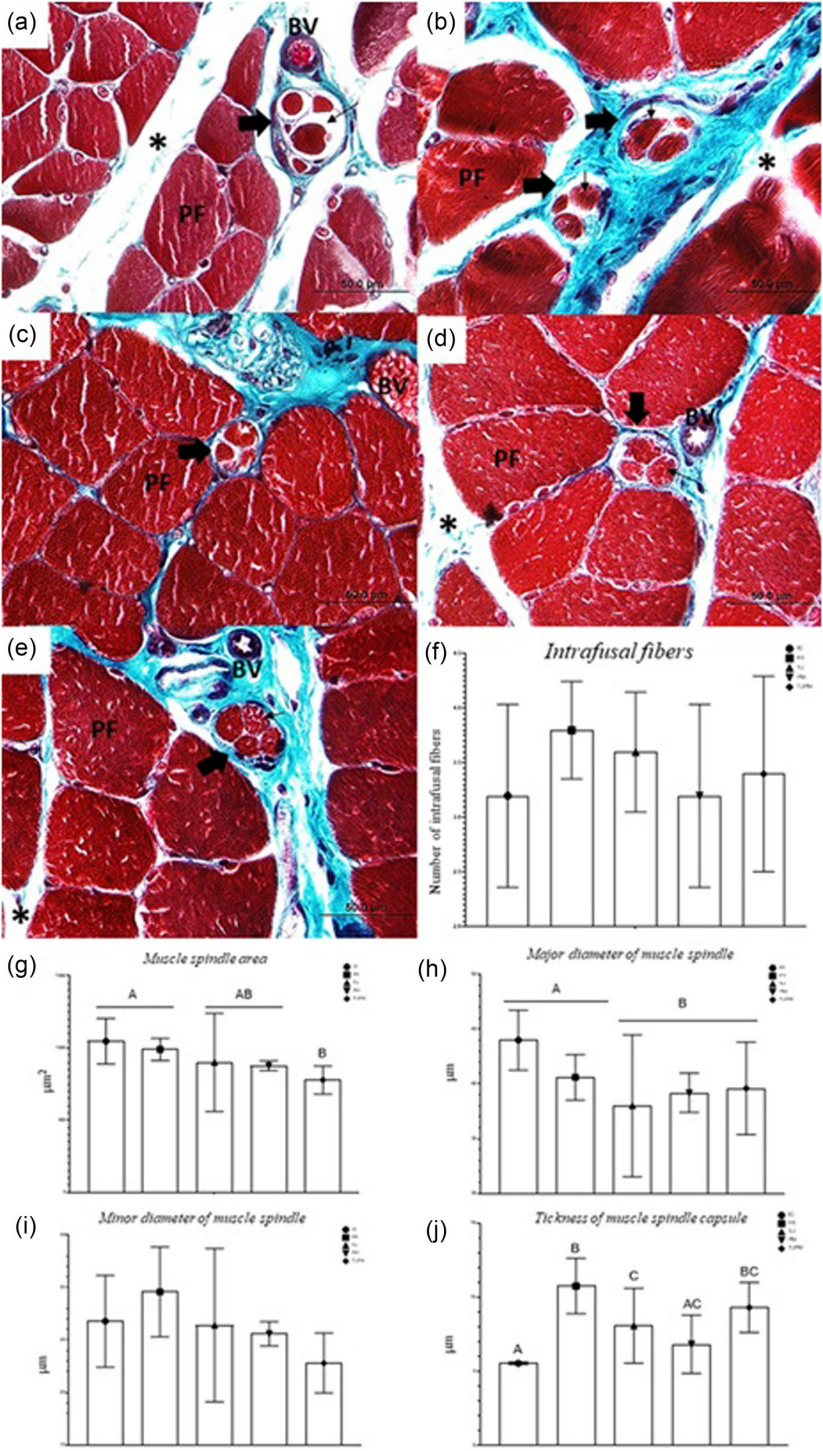
(a–e) Photomicrographs of the soleus muscle from Wistar rats in cross‐section, stained in Masson's Trichrome. (a) Immobilised Group (IG). (b) Free Remobilisation (FR). (c) Passive Mobilisation (PM). (d) Therapeutic Ultrasound (TU). (e) Therapeutic Ultrasound and Passive Mobilisation (TUPM). Are indicated: Spindle capsule (thick black arrow), intrafusal fibers (thin black arrow), polygonal fibre (PF), blood vessel (BV) and connective tissue (*). (f) Number of intrafusal fibers. (g) Muscle spindle area (µm^2^). (h) Major diameter of muscle spindle (µm). (i) Minor diameter of muscle spindle (µm). (j) Thickness of muscle spindle capsule (µm). Different letters represent different values. Values are expressed as mean ± standard deviation (SD). A mixed generalised model test with Fisher (LSD) post‐test was used (*p* = 0.05) (*n* = 8).

In the morphological analysis of muscle spindles, an increase in capsular thickness was observed in the FR and TUPM groups (Figure 5b,e). No major differences were detected among the IG, PM and TU groups (Figure 5a,c,d). Regarding morphometry, no significant differences were observed in the number of intrafusal fibers (*p* = 0.492) (Figure 5f) or in the minor diameter (*p* = 0.101) (Figure 5i). In terms of area (*p* = 0.038), a reduction was observed in TUPM (Figure 5g). For the major diameter (*p* = 0.038), IG and FR were not significantly different from each other (*p* = 0.098) (Figure 5h). In capsular thickness (*p* < 0.0001), higher values were observed in FR, but no difference was detected compared to TUPM (*p* = 0.258) (Figure 5j).

#### Connective tissue estimation

**Figure 6:**
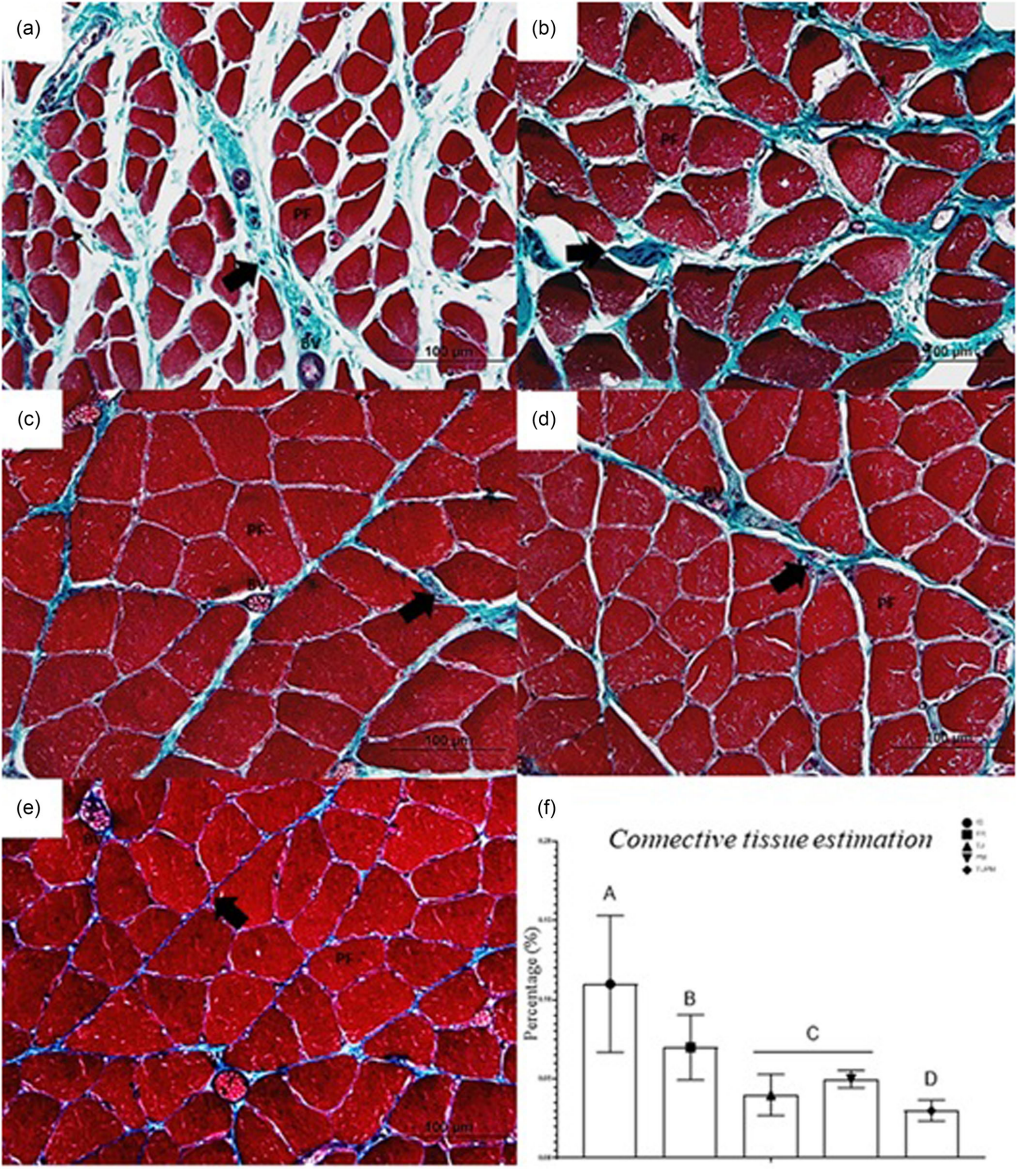
(a–e) Photomicrographs of the soleus muscle from Wistar rats in cross‐section, stained in Masson's Trichrome. (a) Immobilised Group (IG). (b) Free Remobilisation (FR). (c) Passive Mobilisation (PM). (d) Therapeutic Ultrasound (TU). (e) Therapeutic Ultrasound and Passive Mobilisation (TUPM). Are indicated: Connective tissue (thick black arrow), Abnormal fibre (thin black arrow), polygonal fibre (PF), blood vessel (BV). (f) Connective tissue estimation (%). Different letters represent different values. Values are expressed as mean ± standard deviation (SD). A mixed generalised model test with Fisher (LSD) post‐test was used (*p* = 0.05) (*n* = 8).

In the morphology of the connective tissue, large amounts of endomysium and perimysium were observed in the IG and FR groups, which also exhibited marked tissue disorganisation (Figure 6a,b). A reduction in these characteristics was observed in the TU (Figure 6c) and PM (Figure 6d) groups, while the smallest amount of connective tissue and the best tissue organisation were observed in the TUPM group.

When connective tissue density was quantified, the highest value was observed in the IG group (11.2%). The lowest value was observed in the TUPM group (3%) compared to IG, FR, and PM (*p* < 0.0001) and TU (*p* = 0.008) (Figure 6f).

### Analyses performed on cross sections of the soleus muscle stained with Picrosirius red

#### Collagen quantification

**Figure 7:**
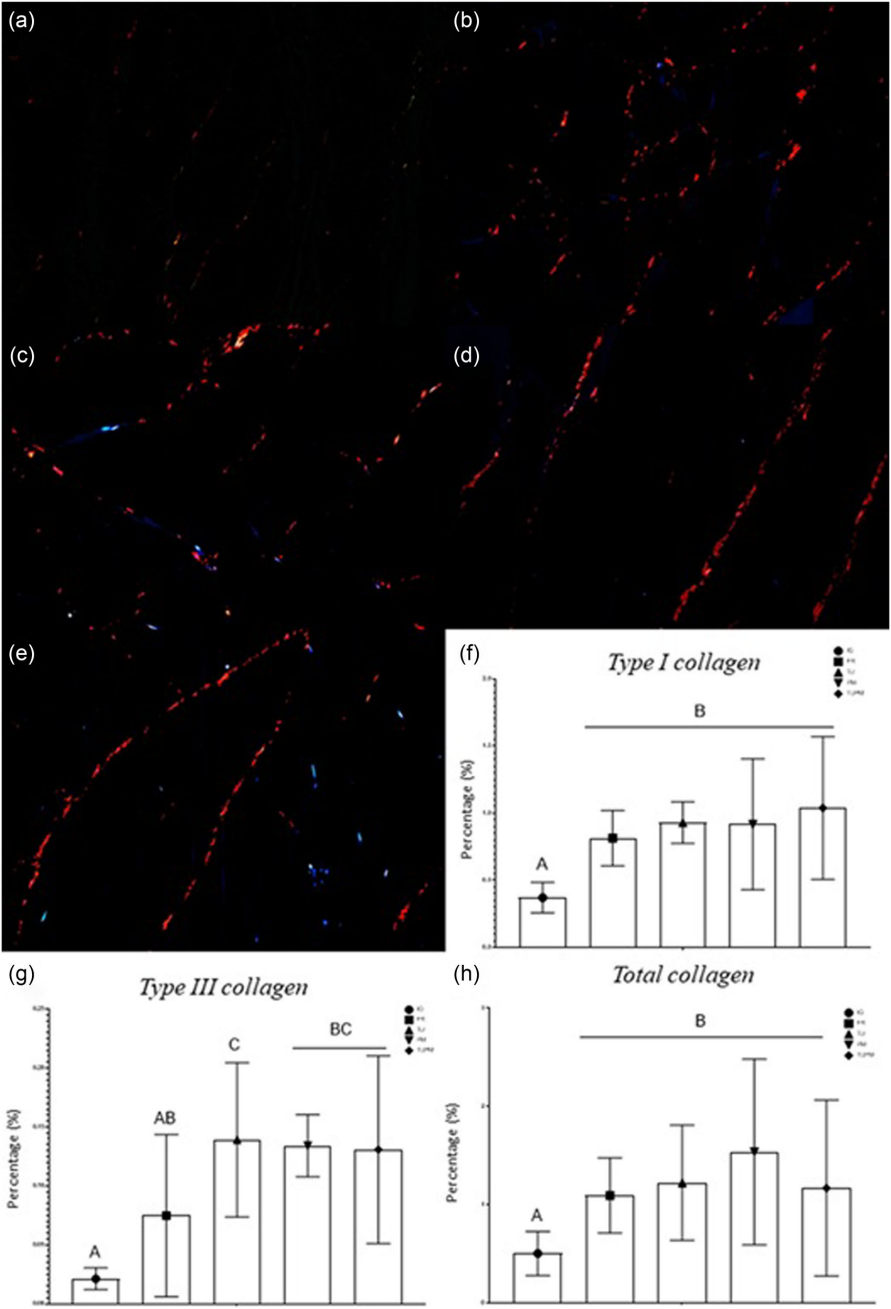
(a–e) Photomicrographs of the soleus muscle from Wistar rats in cross‐section, stained in Picrosirius red. (a) Immobilised Group (IG). (b) Free Remobilisation (FR). (c) Passive Mobilisation (PM). (d) Therapeutic Ultrasound (TU). (e) Therapeutic Ultrasound and Passive Mobilisation (TUPM). (f–h) Collagen quantification (%). (f) Collagen type I. (g) Collagen type III. (h) Total collagen. Different letters represent different values. Values are expressed as mean ± standard deviation (SD). A mixed generalised model test with Fisher (LSD) post‐test was used (*p* = 0.05) (*n* = 8).

In the collagen quantification of the samples, the lowest values of type I collagen were observed in the IG group (Figure 7a–f). Similarly, the lowest total collagen values were detected in IG (Figure 7a–e,h). In the quantification of type III collagen (*p* = 0.0004), lower values were observed in IG. Among the treated groups, TU was the only group that differed from FR (*p* = 0.046) (Figure 7a–e,g).

### Molecular analyses of the plantar muscles—Western blotting

**Figure 8:**
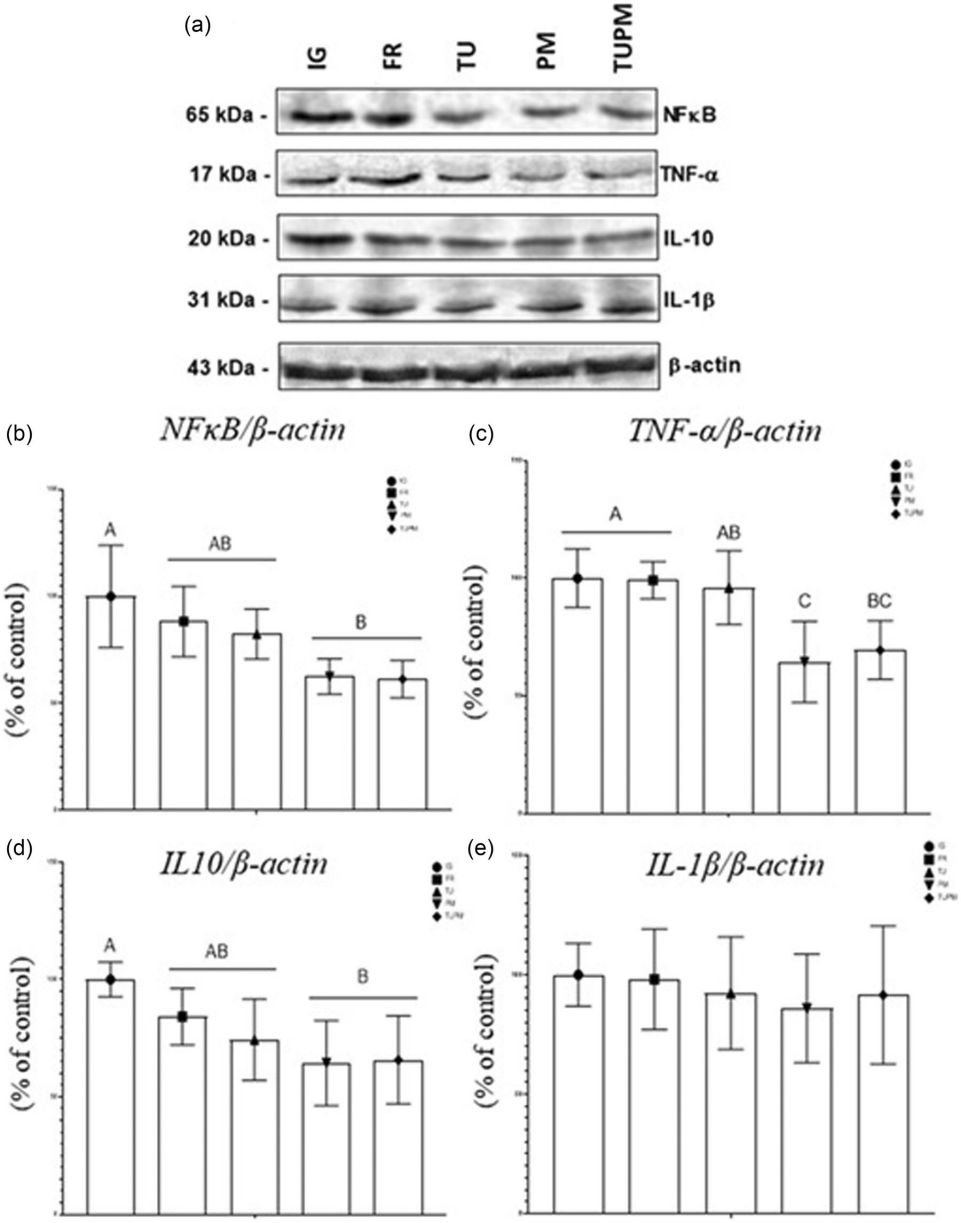
Quantification of cytokines by electrophoresis (% of control). (a) Results expressed on the detection membrane. (b) Nuclear factor kappa beta. (c) Tumour necrosis factor alpha. (d) Interleukin 10. (e) Interleukin 1 beta. Are indicated: immobilised Group (IG), Free Remobilisation (FR), Therapeutic Ultrasound (TU), Passive Mobilisation (PM) and Therapeutic Ultrasound and Passive Mobilisation (TUPM). Asterisk (*) indicates statistical difference (*p* < 0.05). Values are expressed as mean ± standard deviation (SD). A one‐way analysis of variance (ANOVA) was used with Tukey's post‐hoc test (*p* = 0.05) (*n* = 8).

Representative western blot membranes of inflammatory cytokines are shown in Figure 8a. Higher NFκB levels were observed in the IG group (Figure 8b), and similar results were observed for IL‐10 levels (Figure 8d). For TNF‐α, higher values were detected in IG and FR. Within the treated groups, lower TNF‐α values were observed in PM (Figure 8c). No significant differences were detected between groups for IL1‐β levels (*p* > 0.05) (Figure 8e).

## DISCUSSION

The most important finding was that combining TU and PM was more effective in managing the post‐immobilisation period than using either treatment alone. The aim of this study was to a model of immobilisation of Wistar rats, which is advocated in the literature to have effects similar to those observed in humans. Motor function impairment, decreased nociceptive threshold, and muscle hypotrophy in the soleus muscle were induced by 21 days of immobilisation, as indicated by reduced cross‐sectional area and fibre diameters in the group euthanized immediately after cast removal. Inflammatory changes were detected in the immobilisation group, with increased levels of NFκB, TNFα and IL‐10 in the plantar muscle.

### Immobilisation caused damage to functionality and hypotrophy of muscle fibers

During muscle immobilisation, protein synthesis decreases due to reduced stimulation, whereas protein degradation increases, driven by the ubiquitin–proteasome and autophagy–lysosomal pathways [11]. Cross‐sectional area and fibre diameter are directly associated with contraction force, while myonuclei are related to muscle adaptability and remodelling [5]. Reduced rates of muscle protein synthesis have been reported in both humans and animals [10, 16].

Mitochondrial swelling and disintegration are also observed, potentially resulting from the accumulation of autophagic substrates, such as toxic ubiquitinated proteins and abnormal mitochondria. These alterations lead to sarcomere and microtubule disarrangement, muscle weakness and hypotrophy [22, 25]. The accumulation of damaged proteins and altered organelles through autophagy may trigger degenerative processes and exacerbate muscle hypotrophy in immobilised muscles [29], thereby contributing to functional impairment [24]. It should be noted that, in this study, all morphological analyses were performed blindly by the same researcher to minimise bias.

### Greater quantity and disorganisation of connective tissue and less presence of collagen in IG

In this study, the greatest amount of connective tissue was observed in the immobilisation group (IG). Previous studies have suggested that immobilisation induces fibrosis, which may represent a primary cause of contracture damage [33]. Skeletal muscle fibrosis can lead to dysfunctions, such as reduced muscle extensibility [31], ultimately resulting in a limited range of motion [12].

Honda et al. [13] identified two biological processes underlying immobilisation‐induced skeletal muscle fibrosis: the overexpression of transforming growth factor (TGF)‐β1, which promotes connective tissue deposition, and skeletal muscle hypoxia, indicated by elevated levels of hypoxia‐inducible factor (HIF)‐1α. The expression of type I and type III collagen is typically increased in immobilised animals [14]. However, in this study, the lowest collagen levels were found in the IG group compared with the treated groups, possibly due to the application of TU.

TU therapy has been shown to increase collagen concentrations. In a rabbit study, scar cross‐sectional area and type I collagen were increased 6 weeks after injury following low‐intensity TU compared with sham treatment [34]. A systematic review reported that TU facilitated tendon healing, resulting in increased tensile strength and improved collagen alignment [4], and another study demonstrated greater collagen content with enhanced fibre alignment in soft‐tissue lesions [21]. Differences in treatment outcomes for muscle injuries may be attributed to inconsistent descriptions of TU‐treated areas [8].

### Greater thickness of the muscle spindle capsule in FR and TUPM

Since the loss of muscle strength is disproportionate to hypotrophy during periods of immobilisation, it is believed that part of this paresis results from neuromuscular alterations, highlighting the importance of evaluating tissues such as the neuromuscular junction and muscle spindles [6]. An increase in connective tissue was observed with immobilisation, and although no numerical differences in fibers were detected, a reduction in spindle size was observed when subjected to combined therapy. Thickening of the muscle spindle capsule had previously been reported by Mayer et al. [23] in a rat immobilisation model.

Scientific evidence suggests possible links between hypotrophy, muscle spindles, and skeletal muscle functional decline in response to immobilisation [8]. While increased joint movement resistance is often attributed to lesions in central motor pathways, contributions from changes in the passive elastic properties of muscles, connective tissue, tendons and joints have also been observed [20].

Reduced muscle use during immobilisation is believed to result in increased connective tissue growth, as observed in this study, leading to greater passive stiffness of the muscle‐tendon complex. A decrease in stretch reflexes may occur due to reduced muscle spindle responsiveness. Simultaneously, an increase in central gain of the stretch reflex may be induced as an adaptation to reduce reflex circuitry activity. The absence of changes in motor neuron responses to descending activation suggests that this adaptation involves modifications in synaptic transmission efficiency from afferent input to motor neurons, rather than alterations in spinal motor neuron excitability [19].

### TUPM improved the functionality, had the best organisation in the muscle tissue and TU presented an increased vascularisation

It was observed that the combination of TU and PM effectively restored motor function in the IG animals. The high plasticity of striated skeletal muscle tissue allows morphological adaptation to metabolic demands through stimuli such as TU and PM, thereby maintaining functionality [5].

Both thermal and nonthermal effects can be induced by TU, leading to increased tissue temperature, enhanced tissue metabolism, greater collagen fibre extensibility, reduced fluid viscosity and improved local blood flow [28]. Musculoskeletal pain is also reduced by TU through decreased inflammation and promotion of all stages of soft tissue healing. A biostimulatory effect on fibroblasts is exerted by TU, enhancing cell proliferation during muscle degeneration and improving tissue biomechanics in injuries [4].

The results of this study suggest that TU is effective, particularly when combined with PM. This effect may be explained by the crucial role of mitochondria in muscle adaptation [15], as loss of mitochondrial homoeostasis contributes to muscle defects after prolonged disuse [27]. Recovery is accompanied by numerous biochemical and physiological changes, including upregulation of the antioxidant network, mitochondrial biogenesis and enhanced glucose uptake, which supports the nutrition of muscle cells [39].

### Greater presence of NFk β, TNF α and IL‐10 in IG and FR and decrease in PM and TUPM

Prolonged immobilisation is associated with increased proteolysis, oxidative stress, metabolic disturbances, and functional impairments [30], as confirmed in this study. Muscle hypotrophy induced by immobilisation is often characterised as a chronic inflammatory reaction with elevated levels of pro‐inflammatory biomarkers [18]. Pro‐inflammatory cytokines, such as TNF‐α and IL‐6, activate the ubiquitin‐proteasome system, resulting in muscle loss and functional impairment. NF‐κB is also involved in inflammatory responses that may lead to muscle protein degradation, highlighting its role in the regulation of inflammatory processes and protein turnover [32].

Significant reductions in the inflammatory markers TNF‐α and NF‐κB were observed in groups treated with PM and TU combined with PM compared to the IG, FR and TU groups. While passive early motion of the lower limbs has been shown to positively affect inflammation and the immune system [17] its impact on cytokine levels remains less clear. A decrease in IL‐10, an anti‐inflammatory cytokine, and an increase in the pro‐inflammatory IFN‐γ were observed, suggesting that further research is needed [2].

The findings of this study indicate that inflammation is effectively controlled by PM, particularly when combined with TU. This effect is likely mediated by the mechanical field and pulse frequency (1.0 MHz) of TU, which facilitate Ca²⁺ movement, support inflammation resolution, and enhance protein synthesis and tissue restoration [35], thereby positively affecting functional, histomorphometric and inflammatory parameters in musculoskeletal tissue. These results are especially relevant given the significant problems caused by prolonged immobilisation, including degenerative joint changes, synovitis, osteophyte formation, alterations in sensory innervation [9], and loss of muscle strength and function [38]. Considering translational applications for humans, who are often subjected to immobilisation procedures, remobilisation using combined passive mobilisation and therapeutic ultrasound may have considerable potential for promoting recovery.

### Limitations

The limitations of this study include the use of only one dosimetric parameter for both therapeutic ultrasound and the duration and intensity of passive mobilisation. These aspects should be further explored in future studies. Another limitation concerns the muscles selected for analysis, which may not reflect changes in other muscle groups, such as the dorsiflexors. Obviously, in addition to different periods of immobilisation. Despite being an experimental study, the literature shows that the responses of the immobilisation/remobilisation process are similar to those observed in humans, so it is believed that the results obtained here can be an excellent indication of the therapeutic synergy between modalities that can be useful in the recovery process of people subjected to an immobilisation process.

## CONCLUSION

Based on the findings of this study, it can be concluded that limb immobilisation leads to reductions in functional parameters, degeneration of the soleus muscle, and an increased inflammatory response in plantar muscle tissue. The combination of TU and PM therapies produced the most beneficial effects on the analysed parameters. In summary, this synergistic treatment may represent a promising alternative for rehabilitation in individuals who have undergone a period of immobilisation, this may indicate that in humans there should be integration of therapies in post‐immobilisation periods.

## AUTHOR CONTRIBUTIONS

*Conceptualisation*: Fernanda Teixeira Furlan Chico, Ana Caroline Barbosa Retameiro, Aline Reginato, Rafaela Rambo Bremm, Bruna Menegat, Mustafa Munir Mustafa Dahleh, Marina Prigol, Gustavo Petri Guerra, Gladson Ricardo Flor Bertolini and Lucinéia de Fátima Chasko Ribeiro. Methodology: Fernanda Teixeira Furlan Chico, Ana Caroline Barbosa Retameiro, Aline Reginato, Rafaela Rambo Bremm, Bruna Menegat, Mustafa Munir Mustafa Dahleh, Marina Prigol, Gustavo Petri Guerra, Gladson Ricardo Flor Bertolini and Lucinéia de Fátima Chasko Ribeiro. *Formal analysis*: Gustavo Petri Guerra and Gladson Ricardo Flor Bertolini. *Investigation*: Fernanda Teixeira Furlan Chico, Ana Caroline Barbosa Retameiro, Aline Reginato, Rafaela Rambo Bremm, Bruna Menegat, Mustafa Munir Mustafa Dahleh, Marina Prigol, Gustavo Petri Guerra, Gladson Ricardo Flor Bertolini and Lucinéia de Fátima Chasko Ribeiro. *Resources*: Marina Prigol, Gustavo Petri Guerra, Gladson Ricardo Flor Bertolini and Lucinéia de Fátima Chasko Ribeiro. *Writing–original draft*: Fernanda Teixeira Furlan Chico. Writing–review and editing: Fernanda Teixeira Furlan Chico, Ana Caroline Barbosa Retameiro, Aline Reginato, Rafaela Rambo Bremm, Bruna Menegat, Mustafa Munir Mustafa Dahleh, Marina Prigol, Gustavo Petri Guerra, Gladson Ricardo Flor Bertolini and Lucinéia de Fátima Chasko Ribeiro. *Visualisation*: Marina Prigol, Gustavo Petri Guerra and Gladson Ricardo Flor Bertolini. *Supervision*: Lucinéia de Fátima Chasko Ribeiro. *Project administration*: Gladson Ricardo Flor Bertolini. *Funding acquisition*: Fernanda Teixeira Furlan Chico and Gladson Ricardo Flor Bertolini.

## CONFLICT OF INTEREST STATEMENT

The authors declare no conflicts of interest.

## ETHICS STATEMENT

Animal's Use Ethics Committee (CEUA) of the Universidade Estadual do Oeste do Paraná (Protocol no 09‐21, 07/12/2021).

## Data Availability

None declared.
